# Evaluation of range of motion restriction within the hip joint

**DOI:** 10.1007/s11517-012-1016-3

**Published:** 2012-12-22

**Authors:** Glen A. Turley, Mark A. Williams, Richard M. Wellings, Damian R. Griffin

**Affiliations:** 1 WMG, The University of Warwick, Coventry, CV4 7AL UK; 2 University Hospital Coventry, Coventry, CV2 2DX UK; 3 Warwick Medical School, The University of Warwick, Coventry, CV2 2DX UK

**Keywords:** Total hip arthroplasty (THA), Biomechanics, Femoroacetabular impingement, Range of motion

## Abstract

In total hip arthroplasty, determining the impingement free range of motion requirement is a complex task. This is because in the native hip, motion is restricted by both impingement as well as soft tissue restraint. The aim of this study is to determine a range of motion benchmark which can identify motions which are at risk from impingement and those which are constrained due to soft tissue. Two experimental methodologies were used to determine motions which were limited by impingement and those motions which were limited by both impingement and soft tissue restraint. By comparing these two experimental results, motions which were limited by impingement were able to be separated from those motions which were limited by soft tissue restraint. The results show motions in extension as well as flexion combined with adduction are limited by soft tissue restraint. Motions in flexion, flexion combined with abduction and adduction are at risk from osseous impingement. Consequently, these motions represent where the maximum likely damage will occur in femoroacetabular impingement or at most risk of prosthetic impingement in total hip arthroplasty.

**Electronic supplementary material:**

The online version of this article (doi:10.1007/s11517-012-1016-3) contains supplementary material, which is available to authorized users.

## Introduction

Total hip arthroplasty (THA) is one of the most frequently performed reconstructive operations with excellent intermediate to long-term results [17]. However, there are still complications which require the initial procedure to be revised, most commonly due to aseptic loosening and dislocation [45]. Both aseptic loosening and dislocation are associated with not being able to achieve the correct prosthetic component orientation [37]. An overly contained cup may lead to impingement between the neck of the femoral component and the rim of the acetabular cup during terminal motion of the hip. Such contact can create wear particles potentially leading to implant loosening [22, 41, 54]. Further motion beyond the impingement point causes subluxation of the femoral head until the joint dislocates [21, 27, 31]. In contrast, orientating the prosthetic components to maximise range of motion to prevent impingement would mean only partial containment of the hip joint which risks aseptic loosening and joint dislocation whereby the femoral head ‘slips out’ of the acetabular cup [21, 52].

**Fig. 1:**
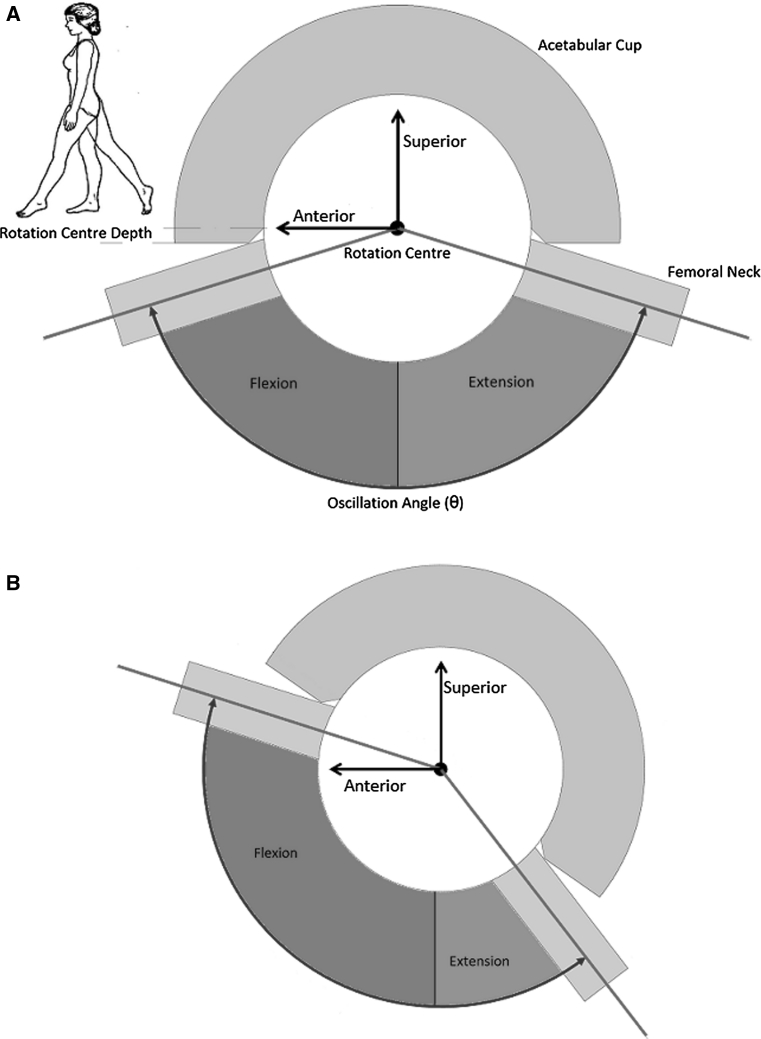
Effect of oscillation angle and component orientation on range of motion in the sagittal plane. **a** Poorly orientated acetabular cup—required amount of flexion cannot be attained within oscillation angle impingement limits. **b** Correctly orientated acetabular cup—required amount of flexion can be attained within oscillation angle impingement limits

Yoshimine and Ginbayashi [57] specified five factors that determine the range of motion which a THA can achieve, four of these are associated with prosthetic component orientation: (1) acetabular cup anteversion, (2) acetabular cup inclination, (3) femoral stem version and (4) femoral component neck axis away from the transverse plane which is dependent upon femoral stem varus–valgus within the femoral canal and femoral component neck-shaft angle [22, 53, 56]. A further orientation parameter has been defined by Renkawitz et al. [36] which is significant in femoral components with a non-axis symmetric neck. It has been defined as ‘femoral tilt’ where the orientation of the femoral neck in the sagittal plane is controlled by where the femoral stem follows the natural anterior bow of the proximal femur [36]. All of these factors interact to affect the position of the hip primary arc of movement, as illustrated in Fig. 1 [52]. The final factor termed the oscillation angle (θ), determines the size of the hip primary arc of movement and is a function of the opening angle of the acetabular liner and the femoral head–neck ratio. Hence, a prosthesis with a large oscillation angle to maximise range of motion and is orientated with good femoral head coverage to achieve a stable joint represents the best balance of these factors [52]. However, increasing the femoral head diameter to maximise the oscillation angle has been shown to increase the risk of femoral neck fracture in hip resurfacing and has been associated with failures in metal-on-metal implants [32, 45]. Therefore, achieving the correct prosthetic component orientation to achieve both ideal range of motion and secure containment within the constrained prosthetic impingement limits is vital to operative success.

Determining the boundary within which an impingement free range of motion is required would not only allow surgeons to determine the optimal prosthetic component orientation in THA but also determine the required bone resection to relieve impingement in a native hip with femoroacetabular impingement (FAI) [1, 49]. At present, specifications for an impingement free range motion outcome have been based on limits of pure joint motion of healthy individuals in the coronal, sagittal and transverse planes [11, 42, 54, 56], or from measuring joint rotations for specific activities of daily living [16, 19, 22, 31]. Other studies have used computer tomography (CT) scans of healthy bony anatomy to determine patient range of motion requirement [23, 46–49]. These studies have acknowledged that range of motion in the native hip is restricted by osseous impingement, soft tissue impingement as well soft tissue restraint. However, these studies have not been able to determine the restriction in range of motion due to soft tissue restraint. Consequently, the aim of this study is to address this gap in knowledge by determining a range of motion benchmark which can identify motions that are at risk from impingement as well as motions which are limited by soft tissue restraint.

## Methods

To identify motions that were at risk from impingement as well as those motions which were limited by soft tissue restraint, data were obtained from literature for 15 activities of daily living of healthy male subjects. These motions were used as the basis to construct a healthy range of motion benchmark which considered range of motion restriction due to both impingement and soft tissue restraint. Range of motion simulation using three-dimensional models constructed from patient CT scans was then used to identify motion restriction due to osseous impingement. Comparing the two motion boundaries we were able to identify those motions at risk of impingement and those which were limited by soft tissue restraint. The 15 activities were—sitting on the floor cross-legged, kneeling with ankles dorsi-flexed and ankles plantar-flexed, level-walking, standing while turning the upper body away, lying supine and rolling over, squatting both with feet flat and on flexed toes, stand-sit-stand from both a normal and a low seat, ascending and descending stairs, standing then bending to retrieve an object from floor, swinging ones leg back and forth, sitting on a normal seat and bending to tie shoe laces, sitting on a normal seat while crossing legs [16, 19, 20, 22, 31]. Using the joint coordinate system developed by Grood and Suntay [14] and adapted for the hip by Wu et al. [55], an anatomical reference frame was constructed with the following axis definitions:

- *x* axis—anterior/posterior axis: abduction/adduction (a)
- *y* axis—superior/inferior axis: internal/external rotation (r)
- *z* axis—medial/lateral axis: flexion/extension (f)

The change in joint angles over the movement cycle has led some researchers to divide a manoeuvre into distinct stages, for example with stand–sit–stand from a normal chair—upright, natural, and leaning forward. For each of the 15 activities, key points were selected by identifying the motions of maximum flexion/extension (f), abduction/adduction (a) and internal/external rotation (r) and then recording the corresponding joint angles at each maximum point in the other two anatomical planes. This produced approximately 50 motion data points for analysis. For each data point, the three-dimensional knee centre positions were calculated using Eq. (1), where the initial knee centre position was defined as position vector $P = (0, - 1,0)$. This position vector accounts for when a person stands in the anatomical neutral posture where they are upright and erect on both legs so that the knee centre position lies directly below the hip centre [26, 39]. This means that the proximal pelvic body segment and the distal femoral body segment are initially aligned so their orientation matrix is a 3 × 3 identity matrix, *I* [5]. Consequently, the orientation matrix $R = \{ [(R_{\text{f}} I)R_{\text{a}} ]R_{\text{r}} \}$can be simplified to define the position of the knee centre using Eq. (1). These knee centre positions were then used to calculate individual axes of rotation for each of the data points by solving Eq. (2), where *R* defines the orientation matrix $R = \{ [(R_{\text{f}} I)R_{\text{a}} ]R_{\text{r}} \}$ and *V* defines the fixed axis of rotation [6, 15, 24]. It was found that 70 % of these manoeuvres occurred about a rotation axis within 15° of the transverse plane indicating the dominant motions for the hip joint were flexion/extension and abduction/adduction coupled with smaller amounts of internal/external rotation [50]. Consequently, rotation axes in the transverse plane were able to be used to simulate range of motion using the CT scans of ten patients for comparison against a healthy range of motion benchmark.

1 $$ P = ([\sin_{\text{f}} \cdot \cos_{\text{a}} ]x,[ - \cos_{\text{f}} \cdot \cos_{\text{a}} ]y,[ - \sin_{\text{a}} ]z) $$

2 $$ QV = [R - R^{\text{T}} ]V = \left[ {\begin{array}{*{20}c} 0 & { - q_{z} } & {q_{y} } \\ {q_{z} } & 0 & { - q_{x} } \\ { - q_{y} } & {q_{x} } & 0 \\ \end{array} } \right] \cdot \left[ {\begin{array}{*{20}c} {v_{x} } \\ {v_{y} } \\ {v_{z} } \\ \end{array} } \right] = \left[ {\begin{array}{*{20}c} 0 \\ 0 \\ 0 \\ \end{array} } \right] $$

### Healthy range of motion benchmark

**Fig. 2:**
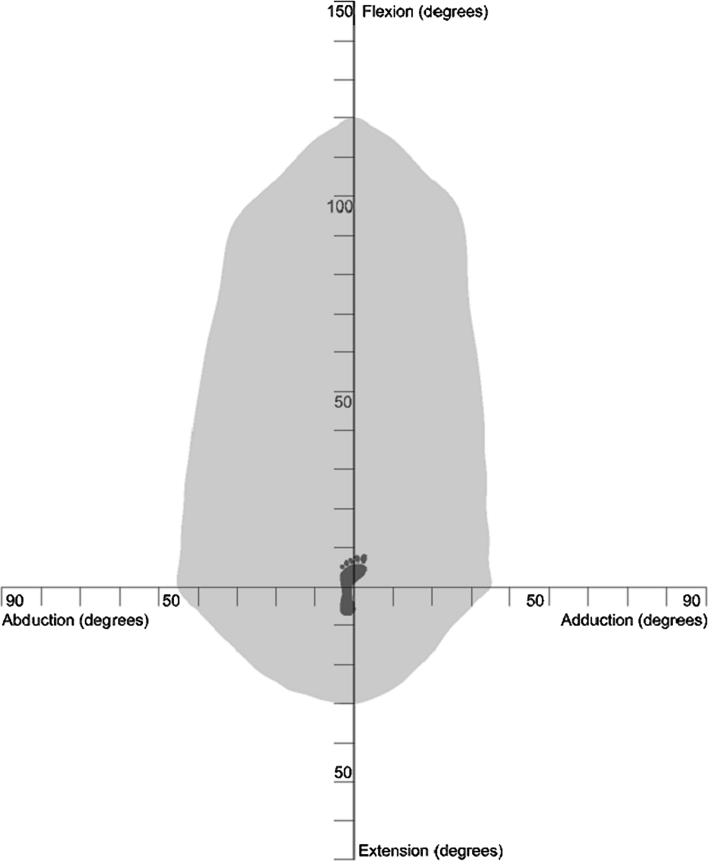
Two-dimensional representation of the healthy range of motion benchmark

**Fig. 3:**
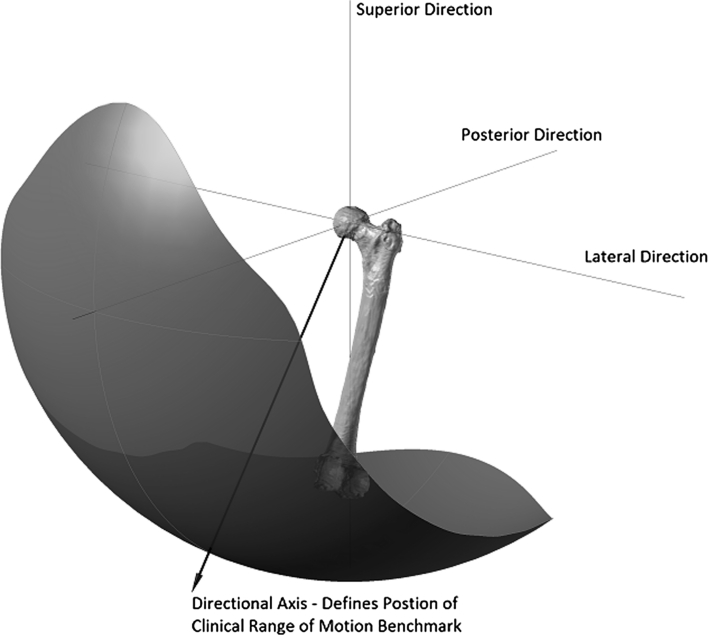
Three-dimensional representation of the healthy range of motion benchmark

To construct a healthy range of motion benchmark, experimental data with regard to pure joint motion of flexion/extension, abduction/adduction and internal/external rotation were used (Table 1) [50]. These pure joint motions have been found to provide a good functional outcome for the patient. Therefore, they correlate positively with being able to perform activities of daily living [10, 19]. Starting again with an initial knee centre position vector $P = (0, - 1,0)$, this vector was rotated about axes in the transverse plane $(T)$ defined in Eq. (3) to simulate daily activities. The angle *α* was rotated about the transverse plane in 10° increments, producing 36 separate rotation axes within this plane. The point at which impingement occurred for each of the transverse plane rotation axes was plotted on both a two-dimensional and three-dimensional plot, which permitted range of motion to be represented graphically as a continuum (Figs. 2, 3). Therefore, the constructed benchmark represented the required range of motion to be achieved to perform daily activities without risk of impingement. Visualisation of how this benchmark was constructed is provided in Additional File 1.

**Table 1:** Reference pure joint motions of the hip [48]

Motion	Angle
Flexion	120°
Extension	30°
Abduction	45°
Adduction	35°
Internal rotation	45°
External rotation	45°

In addition to defining the shape of the healthy range of motion benchmark its position was also defined. To do this, a technique known as moment of inertia analysis was used, where its position was defined using a directional axis (Fig. 3) [9, 13]. The directional axis represented the normal vector to the best-fit plane constructed from points taken at the edge of the range of motion benchmark. The directional axis was determined by calculating the centre of mass of the range of motion benchmark, Eq. (4). The distance of each of the boundary edge points away from the calculated centre of mass was then determined to produce a 3 × *n* matrix, *A*. The dot product $A \cdot A^{\text{T}}$ shown in Eq. (5) was then solved by finding the eigenvector $(\upsilon )$ which maximised the distance to the boundary edge points. Both Figs. 2 and 3 represent range of motion restriction in a healthy hip due to both impingement and soft tissue restraint. The CT method was then used to highlight the impingement risk within the range of motion benchmark.

3 $$ T = (\sin \alpha ,0,\cos \alpha ) $$

4 $$ (\bar{x},\bar{y},\bar{z}) = \frac{{\left( {\sum\nolimits_{n = 1}^{n} {(x)} ,\sum\nolimits_{n = 1}^{n} {(y)} ,\sum\nolimits_{n = 1}^{n} {(z)} } \right)}}{n} $$

$$ {\text{If:}}\quad a_{i} = \sum {x_{i} - \bar{x}} ,\quad b_{i} = \sum {y_{i} - \bar{y}} ,\quad c_{i} = \sum {z_{i} - \bar{z}} $$

5 $$ {\text{Then}}:\quad A \cdot A^{T} = \left[ {\begin{array}{*{20}c} {\sum {a_{i}^{2} } } & {\sum {a_{i} b_{i} } } & {\sum {a_{i} c_{i} } } \\ {\sum {b_{i} a_{i} } } & {\sum {b_{i}^{2} } } & {\sum {b_{i} c_{i} } } \\ {\sum {c_{i} a_{i} } } & {\sum {c_{i} b_{i} } } & {\sum {c_{i}^{2} } } \\ \end{array} } \right] $$

### CT range of motion simulation

For the assessment of range of motion restriction in the hip joint due to osseous impingement, ten CT scans were taken of patients in the supine position. The CT scans used in this study were available on the University Hospitals Coventry and Warwickshire (UHCW) image library. All patients were scanned for clear clinical reasons and informed consent to use their images for teaching and research purposes was obtained. The CT scans used in this study were fully anonymised and performed in accordance with the institution’s ethical guidelines and with the Declaration of Helsinki. The subjects were all male with a mean age of 65.2 years (49–81) and exhibited no evidence of osteoarthritis or abnormal morphology. The scans were acquired on a General Electric LightSpeed CT scanner with a slice thickness of 1.25 mm using a soft tissue algorithm, encompassing the complete anatomy of the pelvis and femur. The CT images in DICOM format were imported into the ImageJ image-processing software (http://rsbweb.nih.gov/ij/). The DICOM scan slices were converted to binary images and a threshold was applied so that only matter with the same density as bone remained. Each slice was then manually cleaned, removing any non-bone material and filling the gaps in the pelvic or femoral trace. Each cleaned image stack was then imported into the Simpleware ScanIP (Simpleware Ltd., Exeter, UK) software package. A morphological smoothing filter set at one pixel spacing was applied to smooth the inconsistencies between slices and a 3D model mesh was then generated for the pelvic and femoral masks. These three-dimensional models were then imported into the Rhino 4.0 NURBS modelling package for use in the motion simulation experiment.

Prior to the range of motion simulation both the pelvis and femur had to be orientated correctly in three-dimensional space. The pelvic coordinate frame was defined using the landmarks of the Transverse Pelvic Plane (TPP) [2]. The medial–lateral axis was defined as a line running parallel to the two anterior superior iliac spines (ASIS) running in the positive direction from left to right with the origin at the hip joint centre. The hip joint centre was defined as the centre of a best-fit sphere of the femoral head. The transverse plane was defined as a plane containing the two ASIS and the mid-point of the two posterior superior iliac spines (PSIS). A line perpendicular to the transverse plane with the origin at the hip joint centre defined the superior-inferior direction. The anterior–posterior axis was constructed orthogonal to the other two axes [55].

The coordinate system of the femur was defined according to the standard defined by Murphy et al. [30]. The superior–inferior axis was defined as running in the positive direction from the knee centre to the hip joint centre. The knee centre was defined by the mid-point of the two femoral epicondyles. The coronal plane was defined as a plane containing the hip joint centre and a line parallel to the posterior aspect of the femoral condyles located at the knee centre. The anteroposterior axis was constructed perpendicular to the coronal plane located at the hip joint centre, and the medial–lateral axis was constructed orthogonal to the other two axes. The femoral 3D model was then aligned so that its axes were coincident with the coordinate frame of the pelvis. The constructed coordinate frame has previously been found to define the neutral rotation of the femur and when aligned with the pelvic coordinate frame forms an orthogonal joint coordinate frame [51]. Consequently, the subsequent range of motion simulation could be directly compared against the healthy range of motion benchmark.

With the pelvis and femur aligned, the Rhino VBScript language was then used to rotate the femur about axes in the transverse plane defined in Eq. (3) and was constrained to 3 DOF with no joint translation [2]. Impingement was deemed to have occurred when the femoral triangle mesh intersected with the rim of the pelvic acetabulum. Once impingement had occurred, no further motion was considered possible. The impingement angle for each rotation axis was plotted on both two-dimensional and three-dimensional plots for analysis, in addition to the CT directional axis. The average range of motion for the ten simulations were plotted and compared with the healthy range of motion benchmark. To define the type of motion restriction—at any point where there was impinged motion of less than 5° between healthy range of motion benchmark and the CT benchmark, then this was defined as osseous impingement, in accordance with the findings by Tannast et al. [48]. A difference between the healthy and the CT benchmarks of less than 10° was defined as soft tissue impingement and any difference greater than 10° was defined as soft tissue restraint.

## Results

**Fig. 4:**
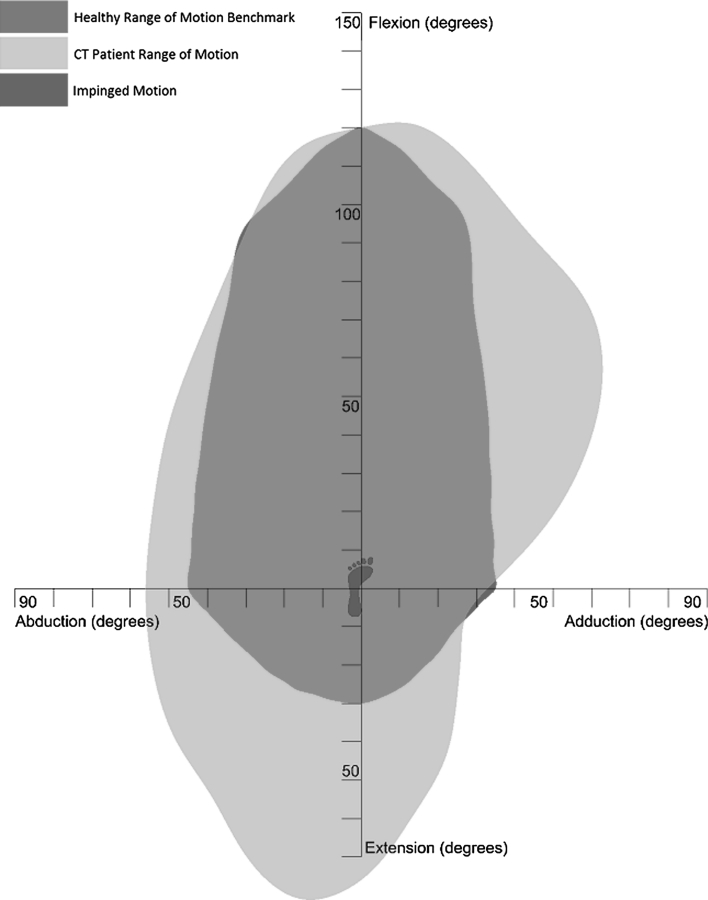
Two-dimensional representation of CT range of motion comparison with the clinical range of motion benchmark

**Fig. 5:**
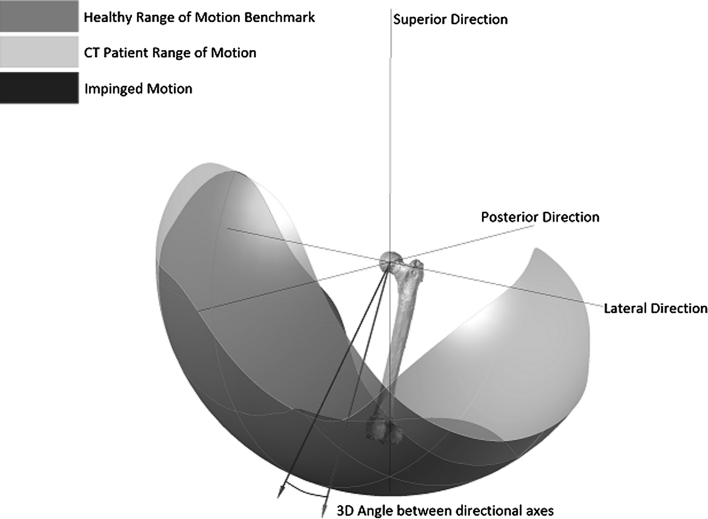
Three-dimensional representation of CT range of motion comparison with the clinical range of motion benchmark

A two-dimensional plot of the CT range of motion (purple) compared to the healthy range of motion benchmark (gold) is shown in Fig. 4. This plot shows that there is osseous impingement present for the motions of adduction, abduction combined with flexion and pure flexion. Impinged motion has been shown in red where the CT range of motion does not encompass the healthy range of motion benchmark. There is a significantly larger CT range of motion when compared with the healthy benchmark with regard to motion in pure extension and adduction combined with flexion, which represented motion limitation due to soft tissue restraint. In all other areas, the CT range of motion is slightly greater than the healthy range of motion benchmark (5°–10°) representing soft tissue impingement. Figure 5 details the three-dimensional comparison of the two experimental results which is also presented in Additional File 1. The directional axis of the CT range of motion (purple) is less elevated than the healthy range of motion benchmark axis (red) and differs by a three-dimensional angle of 15.4°. The two-dimensional angle between these axes in the transverse plane is 3.1°, which disregards the elevation difference between these two axes due to motion in extension. The mean and the standard deviation for the pure joint motions of the 10 CT scans were flexion 120° (σ = 10.2°), extension 77° (σ = 20.1°), abduction 55° (σ = 9.9°) and adduction 33° (σ = 8.8°).

## Discussion

Impingement free range of motion requirement of the hip joint is not well understood. Studies have attempted to quantify range of motion requirement of the native hip through clinical measurements, gait analysis and CT dynamic simulation. These studies have acknowledged that range of motion in the constrained hip is restricted by osseous impingement, soft tissue impingement of the labrum and capsule as well soft tissue restraint [23, 25, 47–49]. No study has yet attempted to fully quantify which motions are associated with these separate causes of motion restriction. This study has provided a comparison of two different methods of analysing range of motion—measurement from previous studies which measured the activities and pure joint motion of healthy individuals which includes all modes of restriction within a person’s motion, and secondly CT dynamic simulation which can identify range of motion restriction due to osseous impingement. Consequently, providing a comparison of the results we were able to distinguish which mode of restriction was limiting range of motion for a given manoeuvre. This was combined with the knowledge that in CT simulations, range of motion restriction slightly over estimates the required range of motion due to the absence of soft tissue by 5° [48].

There are a number of study limitations which should be noted. Firstly, it has been regarded that measurements of healthy individuals between the ages 20–70 provide the most stable and realistic sample from which to base a range of motion benchmark [18, 38]. The CT analysis of hip joint range of motion used patients above this 70 year old threshold. However, this reduction in range of motion is associated with neuro-muscular function rather than morphological changes within the joint and does not affect the result of the CT range of motion experiment [28, 33]. A second limitation was that the two experimental methodologies used data acquired from different subjects. A cadaver study simulating the range motions presented in combination with CT measurement would make the study findings more objective with regard to the effect that soft tissue restraint has upon range of motion [40, 44].

How much the healthy range of motion benchmark can be generalised needs to be considered. This is because the range of motion benchmark has used data from male subjects in its construction. Considering ethnicity, although there may be a greater demand from asian and middle eastern cultures to perform high excursion manoeuvres such as kneeling and squatting [16, 29]. These manoeuvres have been incorporated within the range of motion benchmark and do not exceed the pure joint motion values derived from measurements of mainly European or American subjects. The need for having an alternative range of motion benchmark based on gender is unclear. Data from level-walking studies show that females have in the region of 4°–5° greater motion [3, 4] and similar results were found in a limited number of studies measuring higher demand sporting activities [12, 35]. It is unknown whether this difference transfers across all activities to indicate whether females have greater joint mobility or whether, similar to age, joint excursion is dependent upon other factors such as neurological and muscle interactions. Therefore, it is difficult to assess whether a separate range of motion benchmark is required for a female population.

Analysis of the results with regard to pure flexion, pure adduction and flexion combined with adduction shows that there was impinged motion in these areas (Fig. 4). Impingement in pure adduction was due to collision between the lesser trochanter of the femur with the pelvis. In contrast, impingement in pure flexion and flexion combined with adduction was due to collision between the femoral neck and the acetabular rim in the anterosuperior zone. This is recognised as being the zone in which hip damage occurs, leading to the onset of osteoarthritis [8, 47]. Consequently, motion in this area should be maximised in THA beyond the impingement point to ensure that the femoral neck does not contact with the rim of the acetabular cup. The impinged motion of 90° flexion combined with adduction was also coupled with internal rotation of 33° which the range of motion comparison does not visualise. It is regarded that hip pain due to impingement can be replicated by internally rotating the femur at 90° of flexion [7, 34]. If motion in this position is pain free with 30° of internal rotation then this represents an acceptable range of motion for the hip joint [43]. Consequently, the healthy range of motion benchmark incorporates this amount of normal internal rotation in motions with 90° of flexion. However, knowledge from these previous studies shows that any coupled motion above 90° flexion is a risk area with regard to osseous impingement and should be acknowledged as such [7, 34].

There were a number of motions where the CT range of motion fell within the 5°–10° soft tissue impingement zone defined in Sect. 2.2. Figure 4 shows that abduction is a risk area for soft tissue impingement and is congruent with the findings of Kubiak-Langer et al. [23] and Tannast et al. [47] Therefore, in THA it should be ensured that component positioning ensures that the healthy range of motion benchmark is attained, which for pure adduction is 45°, as this signifies the contact point for soft tissue impingement.

There were two areas where the CT patient range of motion was significantly larger than the healthy range of motion benchmark. These were in the areas of extension, and adduction combined with flexion. It has been found that motion in extension is not limited by osseous impingement; rather it is limited by soft tissue contracture or limitation in secondary joint motion. Consequently, the patient range of motion in this area can be regarded as clinically non-relevant [25]. Analysing Fig. 5, the healthy range of motion directional axis is more elevated than the CT directional axis. If motion in extension is discounted by measuring the two-dimensional angle between these axes in the transverse plane, then the two directional axes align well with only a difference of 3.1°. This demonstrates a close correlation between healthy and CT range of motion, discounting the clinically non-relevant motion in extension and validates the constructed healthy range of motion benchmark.

The extra range of motion found in the area of adduction combined with flexion, when comparing the CT range of motion with the healthy benchmark is a new finding. It is hypothesised two reasons could cause this extreme deviation between the CT range of motion and the healthy benchmark. Firstly, it is not possible to measure true geometrical adduction as a medial rotation in the coronal plane. The opposite leg obstructs the motion. Therefore, measurement of hip joint adduction follows a diagonal motion as the adducted leg is moved in front of the stationary leg. Therefore, the construction of the healthy range of motion benchmark should have considered the pure joint motion adduction benchmark value of 35° in this diagonal plane rather than the coronal plane. Secondly, the extra motion in adduction combined with flexion as exhibited by the CT range of motion may not be limited by osseous impingement. This is because in the CT method, the motion of adduction combined with flexion took the femur into the acetabular notch, permitting extra motion. It is more likely that motion is limited in this area by tension in the adductor muscles. These two hypotheses are a source for further investigation.

**Fig. 6:**
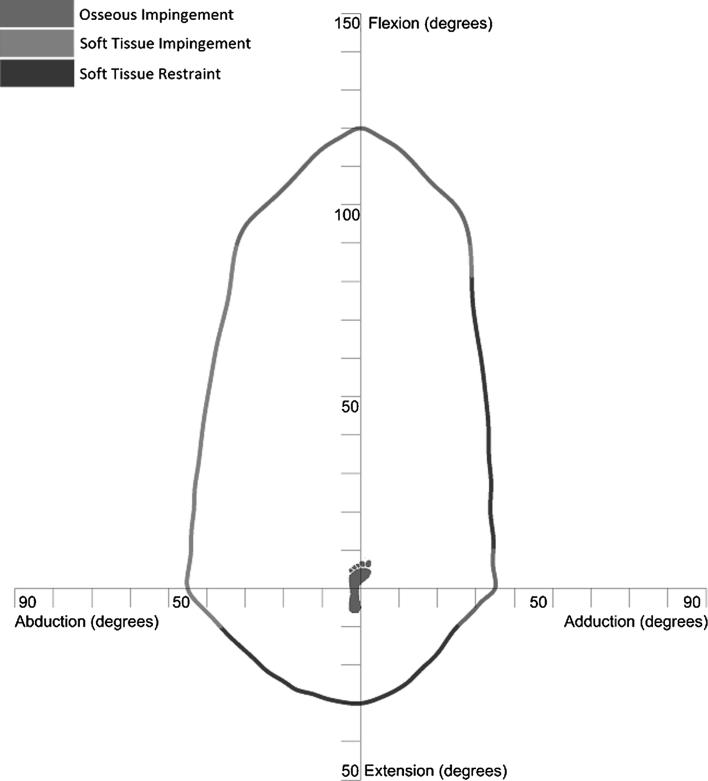
Illustration of range of motion benchmark with impingement zones

This study has used two methods for measuring range of motion in the native hip to determine which factors restrict motion. The results show that motion in pure flexion and flexion combined with adduction are at risk of osseous impingement. These motions represent where the maximum likely damage will occur in femoroacetabular impingement or are at most risk of prosthetic impingement post-THA. The study has also shown that motions in extension and adduction combined with flexion are limited by soft tissue restraint, while motions such as pure abduction are a risk for soft tissue impingement. These separate modalities have been highlighted in Fig. 6 and colour coded to highlight apparent risk—osseous impingement (red), soft tissue impingement (orange) and soft tissue restraint (green). Recognising where the impingement risk is within a person’s range of motion will provide further guidance with regard to where motion is required to be maximised when performing THA or treating femoroacetabular impingement.

## Electronic supplementary material

Below is the link to the electronic supplementary material.

Supplementary material 1 (MP4 12722 kb)
